# Disparities in Hearing Screening Practices in Minnesota Elementary Schools

**DOI:** 10.1002/lary.70566

**Published:** 2026-04-28

**Authors:** Autefeh Sajjadi, Elizabeth Kim, Kathryn S. Marcus, Soorya Todatry, Nicholas Hable, Brianne Roby, Abby C. Meyer, Andrew Redmann, Hannah Herd, Rebecca Maher, Siva Chinnadurai, Amanda Nickel, Asitha Jayawardena

**Affiliations:** ^1^ Department of Otolaryngology‐Head and Neck Surgery University of Minnesota Minneapolis Minnesota USA; ^2^ Department of Otolaryngology‐Head and Neck Surgery University of California San Francisco San Francisco California USA; ^3^ Department of Otolaryngology‐Head and Neck Surgery, Massachusetts Eye and Ear Harvard Medical School Boston Massachusetts USA; ^4^ Department of Otolaryngology Head and Neck Surgery Oregon Health & Science University Portland Oregon USA; ^5^ Department of Internal Medicine Mayo Clinic Rochester Minnesota USA; ^6^ Children's Minnesota, Pediatric ENT and Facial Plastic Surgery Minneapolis Minnesota USA; ^7^ Lions Children's Hearing and Ear, Nose and Throat Clinic University of Minnesota Minneapolis Minnesota USA; ^8^ Children's Minnesota, Pediatric Audiology Minneapolis Minnesota USA; ^9^ Children's Minnesota, Research Institute Minneapolis Minnesota USA

**Keywords:** disparities, early hearing identification, hearing screening, pediatric hearing loss

## Abstract

**Objective:**

Identifying and addressing pediatric hearing loss is critical to supporting a child's development. School‐based hearing screening is a mainstay of timely identification of hearing loss. The objectives of this study were to characterize the current hearing screening practices in public, charter, and private elementary schools in Minnesota.

**Methods:**

This was a cross‐sectional survey with data collected between March and June 2023. Surveys assessed the presence of standardized hearing screening processes and compliance with American Academy of Audiology (AAA) or Minnesota Department of Health (MDH) guidelines.

**Results:**

About 146 public schools, 43 charter schools, and 60 private schools met inclusion criteria and responded to the survey. There was a statistically significant difference in the rate of standardized screening between school types (*χ* ^2^ = 18.06; *p* < 0.001). Only 10.44% (*n* = 26) of schools completed hearing screenings per AAA guidelines, and even fewer, 4.82% (*n* = 12), completed hearing screenings according to MDH guidelines. The odds of screening per AAA or MDH guidelines were 3.50 times higher in charter schools compared to public schools (95% CI: 1.14, 10.73) and 4.61 times higher in private schools compared to public schools (95% CI: 1.73, 12.26).

**Conclusion:**

There is a lack of standardization in hearing screening processes in Minnesota elementary schools. Adherence to screening per AAA or MDH recommendations is low in all types of elementary schools. Charter and private schools were more likely than public schools to screen per AAA or MDH guidelines. Overall, our data demonstrate an opportunity for improvement in school‐based hearing screening.

**Level of Evidence:**

N/A.

## 1 Introduction

Identification and management of pediatric hearing loss is integral to childhood education and development. Undetected hearing loss can impact a child's academic performance as well as their speech‐language, cognitive, and social development [1, 2]. Timely identification of and intervention on hearing loss can improve educational outcomes, language development, and social adjustment [3].

School‐based hearing screening is a mainstay of timely identification of hearing loss after the newborn screen. The standardization of hearing screening has been a public health initiative since the 1970s, but efforts have primarily focused on universal newborn hearing screening [4]. Although the implementation of universal newborn hearing screening has played a major role in increasing equity in pediatric hearing healthcare, no such effort exists for the standardization of school‐based hearing screening. Universal newborn hearing screening is mandated by law and therefore receives congressional funding. However, there is no federal mandate associated with school‐based screening, and there is significant state‐by‐state variability in access to school‐based screening programs [5, 6].

The critical importance of school‐based screenings has been well‐established. Approximately 14% of school‐aged children have transient hearing loss in one or both ears [7, 8]. At age six, only 0.6% of children have permanent hearing loss, but this incidence increases to up to 5% in late adolescence, and can be up to 18% in 18‐year‐olds [7, 9, 10, 11]. Educational outcomes can be impacted with hearing loss in even the mild level thresholds due to compromised speech recognition leading to poorer language development, lower literacy rates, and diminished social skills [12]. Up to 50% of 9‐year‐old children with educationally significant hearing loss underwent newborn hearing screening and were only later identified to have hearing loss [13]. These children who are later identified as having sensorineural hearing loss rely on parental concern (36%) and school‐based hearing screening (32%) to identify this loss [14]. In fact, only 12% of these children were identified through primary care hearing screening [14]. Furthermore, numerous studies have found that standardized school screening programs are cost‐effective interventions for identification of hearing loss [3].

The American Academy of Audiology (AAA) Clinical Practice Guidelines on Childhood Hearing Screening recommend screening children at a minimum in preschool, kindergarten, grades 1, 3, 5, and either 7 or 9 [10]. The Minnesota Department of Health (MDH) recommends annual screening from kindergarten to 3rd grade, and then grades 5, 8, and 11, as well as annual screening for students with chronic or recurrent otitis media, cleft palate/craniofacial anomalies, family history of hearing loss in childhood, and exposure to harmful levels of noise [15]. Both sets of guidelines involve pure‐tone testing at 1000, 2000, and 4000 Hz at 20 dB, with a failed screening if no response at any of these frequencies in either ear [10, 15]. Without federal or state mandates, each public school district or individual private or charter school is responsible for determining their own hearing screening protocol and can heed the recommendations of the AAA and MDH if desired. Public schools within a single school district follow a unified hearing screening protocol determined by the school district.

The purpose of this study is to characterize the current hearing screening practices in elementary schools across Minnesota's two most populous counties (Hennepin and Ramsey County), and in doing so, identify opportunities for improvement. The authors hypothesize that school‐based hearing screening practices and adherence to AAA or MDH guidelines may vary by school type (public, charter, or private) and socioeconomic status.

## 2 Methods

To describe and compare current school hearing practices by school type, we conducted a cross‐sectional survey of elementary schools in Hennepin and Ramsey counties. A list of all registered public, private, and charter schools in 2022 was obtained from the Minnesota Department of Education (MDE). Inclusion criteria for this study were schools with grades 1, 3, and 5, schools with in‐person learning, and schools that were open and functioning at the time of this study. Exclusion criteria were schools that did not include grades 1, 3, and 5, virtual schools, or schools with only online learning. Surveys regarding current hearing screening practices were sent to all eligible schools between March and June 2023 (see Data S1 for full list of questions). Initial surveys were sent to school administrators via email; subsequent follow‐ups for schools that had not responded to email surveys were then conducted via telephone. The surveyor queried school administration who were familiar with hearing screening practices or were directed to the school health office. We defined standardized screening as a hearing screen being performed on all students in at least one grade as opposed to *ad hoc* or specialized screening. Schools that selectively screened only students in special education, individualized education programs (IEP), or per parent/teacher requests were not considered standardized. Compliance with AAA (grades: kindergarten, 1, 3, 5) and MDH (grades: kindergarten, 1, 2, 3, 5) screening guidelines for elementary schools were also assessed for those schools with standardized screening.

We approximated school‐neighborhood opportunity using the US area of deprivation index (ADI) based on census block groups corresponding to each school's nine‐digit zip code. ADI is a measure of geographic socioeconomic disadvantage by factoring in domains of income, education, employment, and housing quality. ADI is scored from 0 to 100, from lowest (0) to highest (100) level of disadvantage and is reported at both the national and state level [16, 17]. We categorized state‐level ADI deciles into low deprivation (deciles < 4), mid deprivation (deciles 4–6), and high deprivation (deciles > 6) areas.

Survey results were summarized using frequencies and percentages. We used Pearson Chi‐Squared test (*χ* ^2^) and Fisher's Exact test, where appropriate, to compare (1) survey responses by school type and (2) school characteristics (type, ADI) by standardized screening practice. To further assess the association of school type, neighborhood opportunity, and standardized screening practice, we employed a logistic regression model adjusted for categorical school type (public, private, or charter school) and ordinal ADI category (low, mid, or high deprivation). Using results from our regression model, we calculated the marginal probability of having a standard screening process based on the distribution of covariates in our sample. Results were reported as odds ratios (OR) and 95% confidence intervals (95% CI). We also used marginal analysis to report predicted probability of standardized screening by school type and ADI category.

This study was submitted to the Children's Minnesota Institutional Review Board (IRB) and was determined to be exempt.

## 3 Results

**FIGURE 1:**
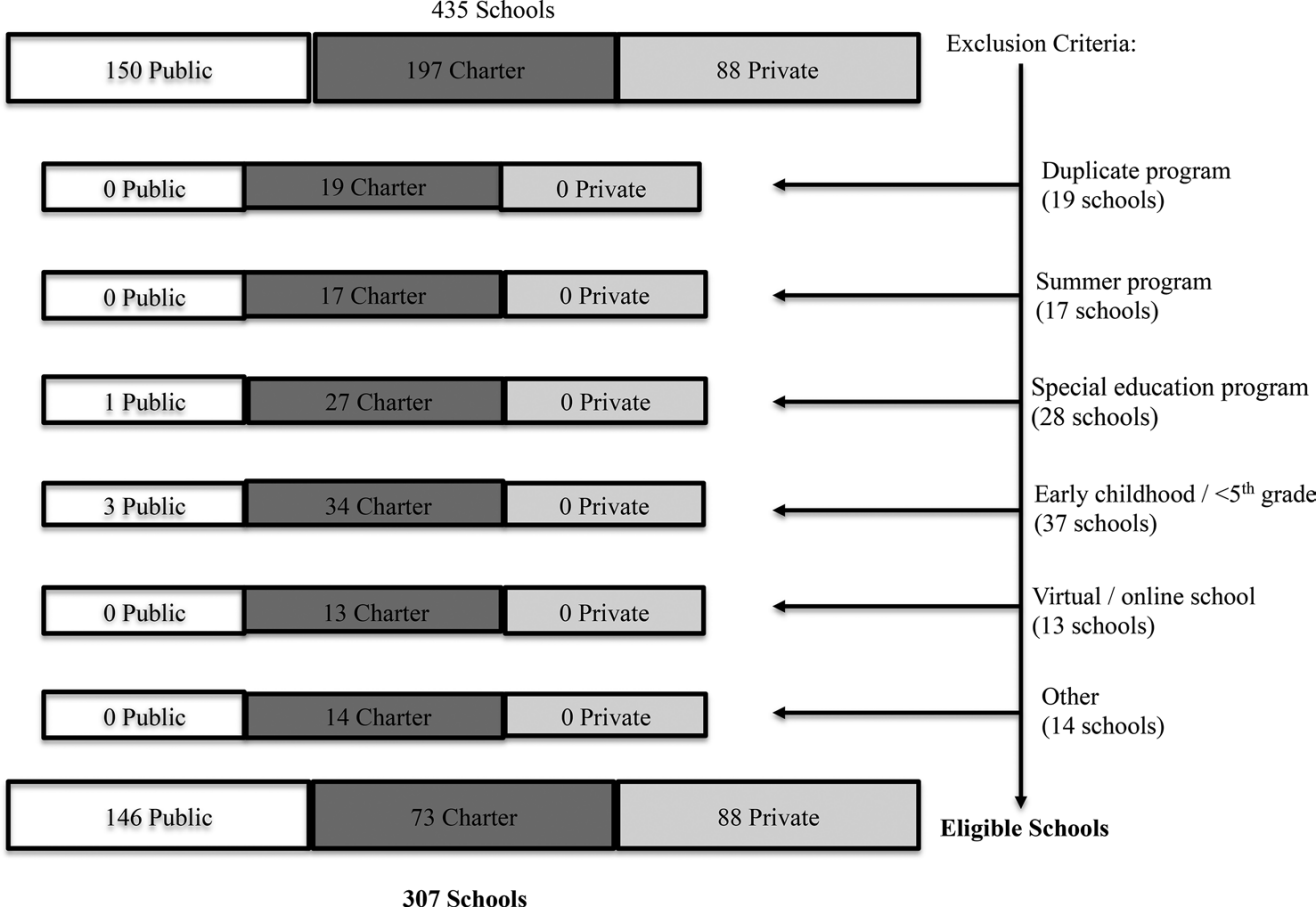
Flowchart of exclusion criteria to determine school eligibility.

The MDE provided a list of all active public (*n* = 150), charter (*n* = 197), and private (*n* = 88) schools with grades 1–5 registered in Hennepin and Ramsey Counties. Ultimately, 307 schools met inclusion criteria, which consisted of 146 public, 73 charter, and 88 private schools (Figure 1). The overall survey response rate was 81.1% (*n* = 249/307), which differed by school type (*χ* ^2^ = 67.1; *p* < 0.001). Survey response rate was 100% for public schools (*n* = 146/146), 58.9% for charter schools (*n* = 43/73), and 68.2% for private schools (*n* = 60/88).

**FIGURE 2:**
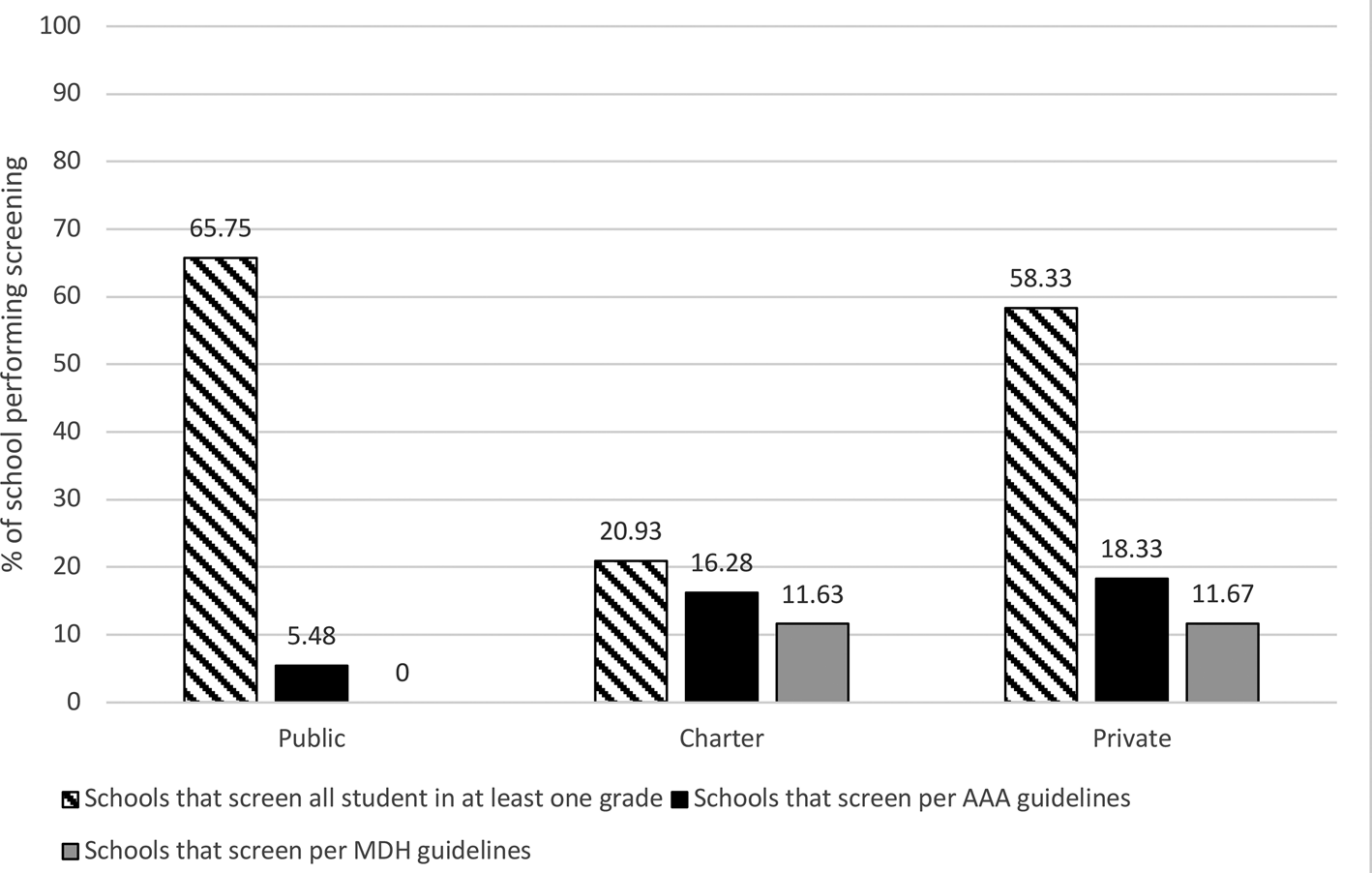
Percent of responding schools that have standardized screening and meet AAA/MDH guidelines.

Survey responses by school type are summarized in Table 1. Among schools that completed the survey (*n* = 249), standardized hearing screening practices for at least one school grade (Kindergarten–fifth grade) were present in 65.75% of public (*n* = 96/146), 32.56% of charter (*n* = 14/43), and 70% of private (*n* = 42/60) schools. There was a statistically significant difference in the rate of standardized screening between public, charter, and private schools (*χ* ^2^ = 18.06; *p* < 0.001). Twelve schools (4.82%) performed hearing screening for all students in all grade levels (five charter, seven private). Conversely, 15 schools (6.02%) performed no hearing screens (5 charter, 10 private). Most schools (56.22%) performed standardized hearing screening for all students in at least one grade. In special circumstances, defined as those students in special education, with an IEP, or parent teacher requests, hearing screens were performed in 34.25% of public (*n* = 50/146), 55.81% of charter (*n* = 24/43), and 13.33% of private schools (*n* = 8/60). Only 10.44% (*n* = 26) of schools completed hearing screenings per AAA guidelines and even fewer, 4.82% (*n* = 12), completed hearing screenings according to MDH guidelines (Table 1, Figure 2). Schools were considered non‐compliant with these guidelines if at least one of the required school grade groups stipulated in their protocols was excluded. There was a statistically significant difference in the rate of screening per AAA guidelines between public, charter, and private schools (*χ* ^2^ = 11.43; *p* = 0.003). There was also a statistically significant difference in the rate of screening per MDH guidelines between public, charter, and private schools (*χ* ^2^ = 19.58; *p* ≤ 0.001). Of the schools who did not complete hearing screenings per AAA or MDH guidelines (*n* = 215), 64.19% were public (*n* = 138/215), 16.74% were charter (*n* = 36/215), and 19.07% were private schools (*n* = 41/215).

**TABLE 1:** Survey responses for hearing screening practices among public, private, and charter schools.

	Public, *n* = 146		Charter, *n* = 43		Private, *n* = 60		Total, *n* = 249		Test^a^	*p*
	*n*	%	*n*	%	*n*	%	*n*	%		
Standardized screening										
Yes	96	65.75	14	32.56	42	70.00	152	61.04	*χ* ^2^ = 18.06	< 0.001
No	50	34.25	29	67.44	18	30.00	97	38.96		
Students screened										
All students	0	0.00	5	11.63	7	11.67	12	4.82	*χ* ^2^ = 65.17	< 0.001
Certain grades	96	65.75	9	20.93	35	58.33	140	56.22		
Special circumstances	50	34.25	24	55.81	8	13.33	82	32.93		
None	0	0.00	5	11.63	10	16.67	15	6.02		
Screen per AAA^b^										
Yes	8	5.48	7	16.28	11	18.33	26	10.44	*χ* ^2^ = 11.43	0.003
No	138	94.52	36	83.72	41	68.33	215	86.35		
Not classifiable	0	0.00	2	4.65	8	13.33	8	3.21		
Screen per MDH^b^										
Yes	0	0.00	5	11.63	7	11.67	12	4.82	*χ* ^2^ = 19.58	< 0.001
No	146	100.00	38	88.37	45	75.00	229	91.97		
Not classifiable	0	0.00	0	0.00	8	13.33	8	3.21		

^a^ Categorical variables compared using Pearson Chi square Test or Fisher's Exact Test for cell sizes < 10.

^b^ “Not classifiable” are schools that did not specify which grades they screen (i.e., “Screen Certain Grades”); not classifiable was not included in statistical comparisons.

Characteristics of schools were compared by standardized screening in Table 2. Of the schools with a standardized hearing screening process (*n* = 152), 42.11% were classified as low deprivation using ADI (*n* = 64/152), 29.61% as mid deprivation (*n* = 45/152), and 26.97% as high deprivation (*n* = 41/152). Two schools were unable to be assigned ADI. Overall, ADI was associated with schools having a standard hearing screening process (*χ* ^2^ = 6.60; *p* = 0.04). The odds of standardized screening were 4.72 times higher among schools in high deprivation areas compared to schools in low deprivation areas after adjusting for school type (95% CI: 1.91, 11.69). Schools in mid and low deprivation areas did not significantly differ in standardized screening practice (OR: 0.97; 95% CI: 0.53, 1.79). School type was also associated with schools having a standardized hearing screening process (*χ* ^2^ = 18.06; *p* < 0.001). Charter schools had 86% lower odds of having a standardized screening process when compared to public schools after adjusting for ADI (95% CI: 0.06, 0.35). There was no significant difference between private and public schools (OR 1.30; 95% CI: 0.67, 2.52).

**TABLE 2:** Comparison of survey responses for standardized hearing screening based on ADI and school type.

	Standardized screening process, *n* = 152		No standardized screening process, *n* = 97		Odds ratio	95% CI	Chi^2^	*p*
ADI	*n*	%	*n*	%				
Low (Deciles 1–3)	64	42.11	45	46.39	Reference	Reference	6.60	0.04
Mid (Deciles 4–6)	45	29.61	37	38.14	0.97	0.53, 1.79		
High (Deciles 7–9)	41	26.97	13	13.40	4.72	1.91, 11.69		
Missing	2	1.32	2	2.06				
School type	*n*	%	*n*	%				
Public	96	63.16	50	51.55	Reference	Reference	18.06	< 0.001
Charter	14	9.21	29	29.90	0.14	0.06, 0.35		
Private	42	27.63	18	18.56	1.30	0.67, 2.52		

Only 26 of 249 total schools completed hearing screening according to AAA or MDH guidelines (Table 3). Of the schools that completed hearing screening per AAA or MDH guidelines, 34.62% (*n* = 9/26) were located in low deprivation areas, 42.31% (*n* = 11/26) in mid deprivation areas, and 23.08% (*n* = 6/26) in high deprivation areas. About 31% of schools that completed hearing screening per AAA or MDH guidelines were public (*n* = 8/26), 26.92% were charter (*n* = 7/26), and 42.31% were private (*n* = 11/26). Eight schools were unable to be classified as following AAA or MDH guidelines due to incomplete information. Overall, ADI was not significantly associated with schools screening per AAA or MDH guidelines (*χ* ^2^ = 1.46; *p* = 0.48); however, school type was associated with screening per AAA or MDH guidelines. The odds of screening per AAA or MDH guidelines were 3.50 times higher in charter schools compared to public schools after adjusting for ADI (95% CI: 1.14, 10.73). Private schools had 4.61 times the odds of screening per AAA or MDH guidelines when compared to public schools after adjusting for ADI (95% CI: 1.73, 12.26).

**TABLE 3:** Comparison of survey responses for screening per AAA or MDH guidelines based on ADI and school type.

	Screen per AAA or MDH guidelines, *n* = 26		Do not screen per AAA or MDH guidelines, *n* = 215		Odds ratio	95% CI	Chi^2^	*p*
ADI	**n**	**%**	**n**	**%**				
Low (Deciles 1–3)	9	34.62	97	45.12	Reference	Reference	1.46	0.48
Mid (Deciles 4–6)	11	42.31	67	31.16	1.69	0.64, 4.43		
High (Deciles 7–9)	6	23.08	47	21.86	1.20	0.37, 3.85		
Missing	0	0	4	1.86				
School type	*n*	%	*n*	%				
Public	8	30.77	138	64.19	Reference	Reference	11.43	0.003
Charter	7	26.92	36	16.74	3.50	1.14, 10.73		
Private	11	42.31	41	19.07	4.61	1.73, 12.26		

**FIGURE 3:**
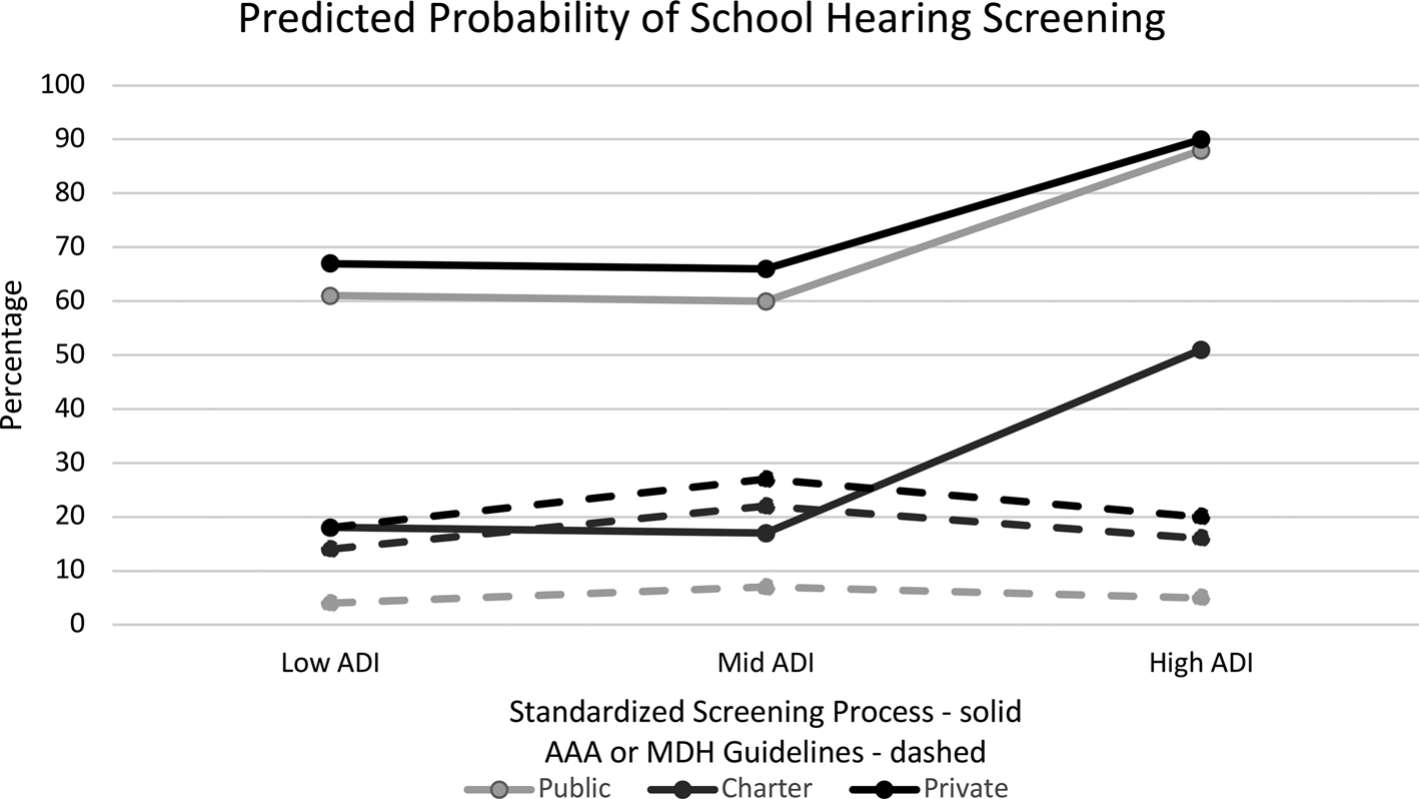
Predicted probabilities of schools having standardized hearing screening and hearing screening per AAA or MDH guidelines according to ADI and school type.

Based on the distribution of characteristics in our sample, we used the results of our logistic regression models to estimate predicted probability of standardized screening (data presented in Table 4 and Figure 3). Private schools in high deprivation areas had the highest probability (90%) of having a standardized hearing screening process (95% CI: 82%, 99%). Charter schools in mid deprivation areas had the lowest probability (17%) of having a standardized hearing screening process (95% CI: 4%, 31%). Private schools in mid deprivation areas had the highest probability (27%) of completing hearing screening per AAA or MDH guidelines (95% CI: 10%, 43%) while public schools in low ADI zip codes had the lowest probability (4%) (95% CI: 1%, 8%).

**TABLE 4:** Predicted probability of standardized hearing screening of the presence of a standardized hearing screening per AAA or MDH guidelines estimated using marginal analysis of the logistic regression models presented in Tables 2 and 3.

	Probability of standardized screening process	95% CI	Probability of screening per AAA/MDH guidelines	95% CI
Public‐low ADI	0.61	0.50, 0.71	0.04	0.01, 0.08
Public‐mid ADI	0.60	0.48, 0.72	0.07	0.01, 0.13
Public‐high ADI	0.88	0.79, 0.97	0.05	0.00, 0.11
Charter‐low ADI	0.18	0.04, 0.31	0.14	0.01, 0.27
Charter‐mid ADI	0.17	0.04, 0.31	0.22	0.05, 0.38
Charter‐high ADI	0.51	0.31, 0.70	0.16	0.03, 0.30
Private‐low ADI	0.67	0.53, 0.81	0.18	0.05, 0.30
Private‐mid ADI	0.66	0.51, 0.81	0.27	0.10, 0.43
Private‐high ADI	0.90	0.82, 0.99	0.20	0.03, 0.38

When asked if schools would be interested in participating in a pilot program expanding the current screening practices with free hearing screens for students in grades 1, 3, and 5, an overwhelming majority (88.4%, *n* = 220/249) of schools expressed some interest (*n* = 83, yes; *n* = 137 maybe interested). Schools that were in highly disadvantaged areas were more likely to be interested in participating in the pilot program to expand screening practices (94.4%) compared to mid (85.3%) and low (85.3%); yet this was not statistically different (*χ* ^2^ = 2.90; *p* = 0.24). Public schools were significantly more likely to express interest (98.6%) compared to charter (86.1%) and private schools (65%) (*χ* ^2^ = 47.0; *p* < 0.001). Schools were equally likely to be interested in the pilot program regardless of if they already had standardized screening practices (*χ* ^2^ = 0.87; *p* = 0.35).

## 4 Discussion

The incidence of hearing loss increases from 0.6% at age 6 to up to 5% in late adolescence [9, 10]. There are myriad potential etiologies for progressive pediatric hearing loss including infection, trauma, exposure to ototoxic medication, progressive congenital genetic disease, congenital cytomegalovirus, and noise exposure [11]. A recent report by the World Health Organization suggested that 1 billion adolescents are at risk for hearing loss due to unsafe listening practices, a trend that is only increasing with increased access to smartphones in children [18]. Delayed or undiagnosed hearing loss is associated with listening and communication difficulties, language and speech delays, reduced school performance, lack of career planning and success in the workplace, social isolation, higher rates of depression, and lower quality of life [19]. School‐based hearing screening offers a unique opportunity for identification, diagnosis, and management of children who might suffer from hearing loss, and particularly for those with limited access to primary care. The American Academy of Pediatrics recommends objective hearing screening at the four‐year well child check; however, the rate of attendance of this visit is 72% [20, 21]. Over 95% of children of elementary school age are enrolled in public, private, or charter schools, while far fewer have reliable access to primary care [22]. In fact, a 2016 report suggested that 20.3 million children (28%) have limited access to a pediatrician [23]. School‐based screening, therefore, serves a critical role in evaluating ear and hearing health of children lost to follow up after referral following newborn hearing screening as well as those with progressive hearing loss, regardless of access to healthcare outside of school.

The present study demonstrates a widespread lack of comprehensive school‐based hearing screening practices for children in Hennepin and Ramsey counties in Minnesota. Only 56% of respondents performed standardized hearing screening for all students in at least one grade, and of these schools, only 10% (*n* = 26) performed hearing screens consistent with the AAA Clinical Practice Guideline for elementary schools. Even fewer schools, 5% (*n* = 12), met MDH guidelines. No public schools in Hennepin or Ramsey counties completed hearing screening according to MDH guidelines. The notable difference in the rate of standardized hearing screenings in at least one grade compared to the minimum of four or five grades required by AAA or MDH guidelines, respectively, suggests an overall willingness to engage with school‐based hearing screening, but perhaps a lack of resources or staffing to allow for grade‐wide screening across multiple grades. Public schools were the least likely, and private schools were most likely, to complete screening per AAA/MDH guidelines. While most schools did seem to understand the benefit of performing some form of standardized screening, these findings underscore a need for accessible, standardizable, and affordable elementary school hearing screening in Minnesota schools. Encouragingly, most respondents expressed strong interest in expanding their programs.

The differences in adherence to AAA or MDH guidelines between public versus private or charter schools may also reflect differences in school policy governance, organizational structures, and accountability. Without federal or state mandates, each school is responsible for determining its own hearing screening protocol. This can happen at a more individualized level for private and charter schools. All public schools within a school district have a unified hearing screening protocol determined by the district. It is possible this results in less agency for individual public schools to increase the number of grades in which annual hearing screening is completed. If protocol changes are suggested at a district, rather than individual school, level, it may also take longer for these changes to be disseminated and implemented.

This study utilized ADI of schools' locations as a proxy for variables related to socioeconomic status. We hypothesized that schools in areas with higher levels of disadvantage would be more vulnerable to hearing screening disparities. Our data demonstrated that schools in areas of the highest levels of disadvantage were, in fact, more likely to have standardized hearing screening processes. ADI was not significantly associated with schools screening per AAA or MDH guidelines. This provides some encouragement that access to hearing screening may not be negatively impacted by attending school in more socioeconomically disadvantaged zip codes. However, children from families with lower socioeconomic status may be at greater risk for loss to follow‐up after being identified during screening and may require additional resources to facilitate next steps in care [24]. Thus, while access to screening itself may not be impacted by ADI, post‐screening practices and referral pathways may not be equivalent between groups. Future study of disparities in access to post‐hearing screening care and follow‐up is warranted.

This study has a number of limitations that are important to understand when interpreting these findings. Public schools had a 100% response rate, while charter and private schools responded at a lower rate (59% and 68%, respectively). This may result in a selection bias; schools that did not perform screenings may have been less likely to report these findings. Conversely, schools that were not concerned about hearing screening resources may have been less likely to respond. The overall response rate of 81% in our survey limits this selection bias. As we relied on schools to answer the phone or email, schools with limited resources may not have had the staff to participate in the survey, which may suggest a participation bias. Our ADI calculation is based on the zip code of a school, not on the zip codes of students. For public schools, the ADI may be more representative of the students attending the school, whereas students attending private schools may come from families with greater flexibility to choose a school in a different zip code than their home zip code. This study only reports data from the two most populous counties in Minnesota, and additional research is necessary to understand this trend in more rural regions. Further research is also needed to understand the generalizability of our findings in other states. There is a paucity of literature on hearing screening completion rates nationwide after the newborn period [25]. The School Health Policies and Practices Study, a CDC national survey from 2014, reports that 77.2% of schools conduct hearing screening; however, there is no further specific description of which grade levels or how many grade levels are screened [26]. Yong et al. convey both the scarcity of guidelines for school hearing screening practices and the inconsistent implementation of the few guidelines that exist [25]. Barriers in reporting data regarding standardized hearing screening practices across the country include the lack of a centralized surveillance system for tracking hearing screening rates and variable hearing screening policies across states.

## 5 Conclusion

School‐based hearing screening presents a critical opportunity for identification of, and intervention upon, pediatric hearing loss. In the two largest counties in Minnesota, only approximately half of all schools perform hearing screenings of at least one school grade, and far fewer follow screening guidelines set by the AAA or the MDH. When they had a standardized screening process, private schools were more likely to screen according to AAA/MDH guidelines compared to charter or public schools. These findings underscore a need for increased access to, and improvement in, standardized school‐based hearing screening protocols.

## Funding

The authors have nothing to report.

## Disclosure

Abstract ID: 1578469; this abstract was presented at the AAO‐HNSF 2023 Annual Meeting & OTO Experience, Nashville, Tennessee, October 1–4, 2023.

## Ethics Statement

This study was found to be IRB exempt.

## Conflicts of Interest

The authors declare no conflicts of interest.

## Supporting information


**Data S1:** Survey questions regarding hearing screening practices of eligible schools.

## Data Availability

The data that support the findings of this study are available from the corresponding author upon reasonable request.
