# Supplementary material for: Disparities in Hearing Screening Practices in Minnesota Elementary Schools
Source: Laryngoscope. 2026 Apr 28;136(9):4064–71. doi: 10.1002/lary.70566 (PMC13460717; doi:10.1002/lary.70566)
Supplement: Supplementary file 1 [file LARY-136-4064-s001.docx]

**Supplementary Material S1.** Survey questions regarding hearing screening practices of eligible schools.

1. Is there a standardized hearing screen at your school (defined as all students in at least one grade are screened)?
2. Which students get a hearing screen?
3. Who administers the hearing screen?
4. Do you have a dedicated testing room?
5. Have the noise levels in the room used for hearing screens been tested?
6. Would you be interested in participating in a pilot program to expand and enhance hearing screening at your school?
